# Developing a Standardised Dataset for Natural History Studies in Fibrous Dysplasia/McCune-Albright Syndrome

**DOI:** 10.1007/s00223-025-01379-5

**Published:** 2025-05-02

**Authors:** Ana Luisa Priego Zurita, Oana O. Bulaicon, Jillian Bryce, Nerea Arrieta, Magdalena Caballero Campos, Mariya Cherenko, Gaby Doxiadis, Corinna Grasemann, M. Kassim Javaid, Helen McDevitt, Stijn W. van der Meeren, Diana Ovejero Crespo, Luisa de Sanctis, Lothar Seefried, Annemarie A. Verrijn Stuart, Daniele Tessaris, Pieter Bas de Witte, Roland Chapurlat, S. Faisal Ahmed, Natasha M. Appelman-Dijkstra

**Affiliations:** 1 Department of Internal Medicine, Division of Endocrinology, Leiden University Medical Center, Albinusdreef 2, Post box 9600, 2300 RC Leiden, The Netherlands; 2 University of Glasgow, Office for Rare Conditions, Glasgow, United Kingdom; 3 Asociación de Displasia Fibrosa, Bergara, Spain; 4 Department of Mathematics, University of Cordoba, Cordoba, Spain; 5 Patiëntenvereniging Fibreuze Dysplasie, Nijkerk, The Netherlands; 6 Department of Paediatrics-Katholisches Klinikum Bochum, Ruhr-University Bochum, Bochum, Germany; 7 Nuffield Department of Orthopaedics, Rheumatology and Musculoskeletal Sciences, University of Oxford, Oxford, United Kingdom; 8 Paediatric Bone and Endocrinology, Royal Hospital for Children Glasgow, Glasgow, United Kingdom; 9 Department of Ophthalmology, Leiden University Medical Center, Leiden, The Netherlands; 10 Centro de Investigación Biomédica en Red de Fragilidad y Envejecimiento Saludable (CIBERFES), IMIM (Hospital del Mar Research Institute), Barcelona, Spain; 11 Pediatric Endocrinology, Regina Margherita Children Hospital-Aou Città Della Salute E Della Scienza, Torino, Italy; 12 Orthopaedic Department, University of Würzburg, Würzburg, Germany; 13 Department of Pediatric Endocrinology, Wilhelmina Children’s Hospital, University Medical Center Utrecht, Utrecht, The Netherlands; 14 Department of Orthopaedics, Leiden University Medical Center, Leiden, The Netherlands; 15 INSERM UMR 1033, Université Claude Bernard-Lyon 1, Service de Rhumatologie, Chu Edouard-Herriot, Lyon, France; 16 Developmental Endocrinology Research Group, School of Medicine Dentistry & Nursing, University of Glasgow, Glasgow, United Kingdom

**Keywords:** Fibrous dysplasia, McCune-Albright syndrome, Rare bone disease, Registries, Data collection

## Abstract

Fibrous dysplasia/McCune-Albright syndrome (FD/MAS) is a rare and complex condition caused by somatic variants in the *GNAS* gene that lead to a wide clinical spectrum. The diagnostic process and therapeutic pathway vary per centre and therefore international harmonisation of data collection should be pursued. To understand the diagnostic pathways and clinical outcomes of patients with FD/MAS reported on an electronic-reporting tool (e-REC) across European centres to guide the develop a condition-specific module within the European Registries for Rare Endocrine and Bone conditions. Centres that reported new cases on e-REC between October 2019 and May 2021 were approached to complete a survey in May 2021. Fifty-eight cases were included. Median age at presentation was 20 years (range, 0, 72). Of the 58 included cases, the presentation type was isolated craniofacial FD in 19 (33%), monostotic FD in 15 (26%), polyostotic FD in 10 (17%), and MAS in 13 (13%). Standardised questionnaires to assess pain and quality of life were used routinely in 21/58 patients (36%). The majority of patients had more than one healthcare provider, with great diversity in the specialty of the coordinating physician. A standardised dataset module for FD/MAS was developed through collaboration with the FD/MAS study group, incorporating expert consensus and clinical insights. Key variables were identified to capture essential diagnostic, clinical, and patient-reported outcomes. The diagnostic path for patients with FD/MAS across European expert centres is variable. The outcomes of this study allowed the building of the first international FD/MAS-specific data collection.

**Supplementary Information:**

The online version contains supplementary material available at 10.1007/s00223-025-01379-5.

## Introduction

Fibrous dysplasia/McCune-Albright syndrome (FD/MAS) is a rare disorder (estimated prevalence between 1/100,000 and 1/1,000,000 [1, 2]), caused by a post-zygotic gain of function somatic variant in the *GNAS* gene leading to a broad clinical spectrum. FD/MAS is a mosaic condition that can occur either as single skeletal lesions (monostotic disease), as multiple skeletal lesions (polyostotic disease), as an association of skeletal disease with extra skeletal features such as hyperfunctioning endocrinopathies, benign tumours and hyperpigmented skin lesions known as McCune-Albright syndrome (MAS), or as a combination of skeletal lesions and myxomas, known as Mazabraud Disease [3]. Moreover, the presence of two or more extra skeletal features without FD is enough to diagnose MAS. Patients can present with either asymptomatic isolated or multiple skeletal lesions or associated with deformities, pain and fractures [3, 4]. Endocrinopathies described in patients with FD/MAS include neonatal hypercortisolism, precocious puberty due to gonadotropin-independent sex steroid production, non-autoimmune hyperthyroidism, autonomous growth hormone excess and hyperprolactinaemia.

Patients with FD/MAS often experience significant pain, which can negatively impact their quality of life [4, 5]. Pain severity is not influenced by FD subtype, and pain scores may be similar in patients with monostotic lesions compared to those with severe disease extension [6, 7]. Currently, there is no standard treatment for FD; analgesics and off-label bisphosphonates are prescribed widely to alleviate pain. While oral bisphosphonates have not been proved to alleviate pain, intravenous bisphosphonates have been shown to reduce pain and are recommended for treating persistent moderate to severe pain [8, 9]. Denosumab, a humanised monoclonal antibody, has been used off-label as well, since it has been shown to successfully reduce pain and lesion extension [2, 10–13]. The lack of uniform diagnostic approach as well as long-term monitoring of these patients has led to potential misdiagnosis and treatment [14, 15].

**Fig. 1:**
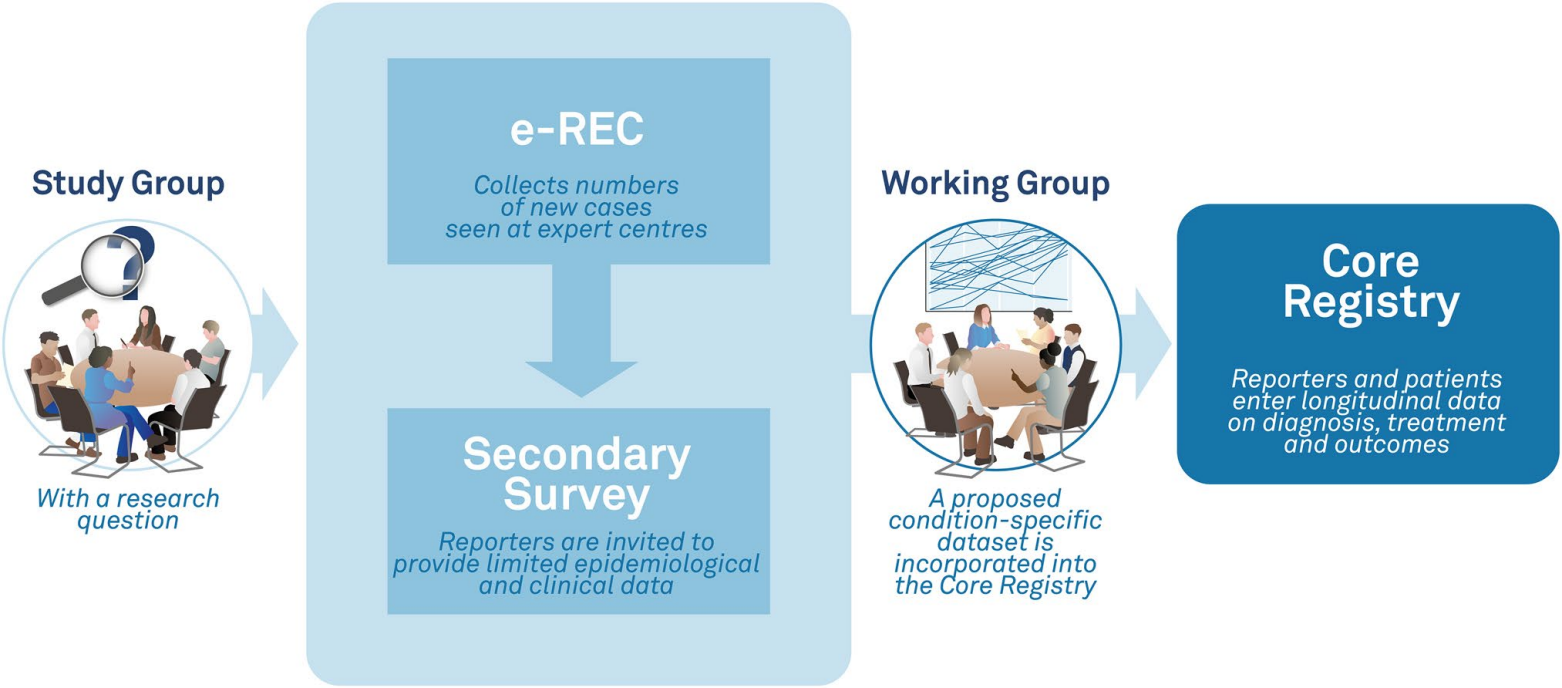
Dual-platform approach to rare disease research within The European Registries for Rare Endocrine and Bone conditions (EuRREB). The e-reporting tool (e-REC) is used to map centres caring for rare conditions. Upon reporting a case, a unique ID is generated for each case and provided electronically to be stored at the reporting centre. Reporters of the condition of interest are invited to complete a survey collecting routine clinical data. The expertise of the Working Group together with the results of the survey are used to develop a set of variables that are built into the Core Registry with the purpose of collecting condition-specific outcomes

Natural history registries that collect clinician and patient-reported outcomes can address knowledge gaps and assist in developing best practice guidelines to improve patient outcomes in rare conditions such as FD/MAS. To date, two patient-driven international registries, the FD/MAS Patient Registry and the FD/MAS Alliance, have been collecting data on FD/MAS. However, a standardised clinical dataset is lacking. To support the needs of healthcare providers, patients and researchers linked to the European Reference Networks for Rare Bone Diseases (ERN BOND) and Rare Endocrine Conditions (Endo-ERN), a joint platform, the European Registries for Rare Endocrine and Bone Conditions (EuRREB) has been developed. During the design phase, a stakeholder group comprising multidisciplinary expertise and patient advocacy representatives was formed to assess current practices and develop a FD/MAS condition-specific module for collecting patient and clinician-reported outcomes within the registry platform (Fig. 1).

## Methods

The EuRREB platform consists of two registries, an e-reporting tool (e-REC) and a Core Registry. The former acts as a surveillance platform that collects the number of new cases of rare conditions encountered at a centre in each month, but does not collect any personal identifiers and does not require patient informed consent. The Core Registry can collect core information on several endocrine and bone conditions in its core dataset and detailed information about specific conditions in its condition-specific modules (CSM). As the Core Registry collects personal identifiers, informed consent from the patient is needed. Furthermore, the development of a CSM requires input from experts within the field. The project complies with EU GDPR and was approved by the Information Governance authorities at the NHS Greater Glasgow & Clyde Health Board and the National Research Ethics Service in the UK (e-REC REC Reference 17/WS/0178; Core Registry REC Reference 18//WS/0205) and later at the Leiden University Medical Center (e-REC project ID 133036; Core Registry protocol nWMODIV2_2024018, project ID 130850).

For the present study, following notification of a case on the e-REC platform, the reporting clinician was invited to complete a clinical outcomes questionnaire to understand the natural history and diagnostic pathway of the case. The questions collected data regarding the presentation and diagnosis, healthcare professionals involved, treatment, monitoring and follow-up. The questionnaire was designed by the registries management team with input from the FD/MAS expert group and distributed using Webropol (Valiant Office Suites, Valley Drive, Rugby, UK). Thirty-two questions were developed including a combination of numerical, multiple-choice, matrix and open and close-ended questions (Online Resource 1). Between October 2019 and May 2021, 80 new cases of FD/MAS were reported on e-REC by nine centres in five European countries, with a median of four cases per centre (range 1–54). The survey was open between May and July 2021. Qualitative variables were described with their median and range; qualitative variables were described by their frequency. Microsoft™ Excel™ 2019 (Microsoft Corp, Redmond, WA, USA) and GraphPad Prism version 10.2.3 were used for data extraction, data analyses, and graphical presentation of the results.

## Results

### Survey Results

#### Description of Cases

**Fig. 2:**
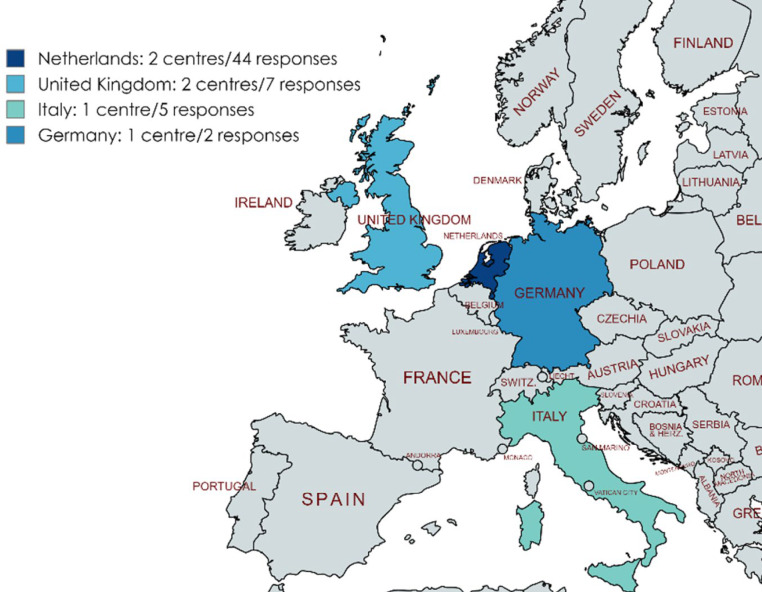
Countries participating in the study and their contribution. Image created using Mapchart.net

Seven clinicians from six centres completed the questionnaire for 67/80 reported cases (response rate 84%) (Fig. 2). The status of the diagnosis FD/MAS was *confirmed* in 58/67 cases (87%), in four *suspected* (6%) and in five (7%) *excluded*.

Table 1 shows the clinical and demographic characteristics of patients with confirmed FD/MAS. The most frequent disease subtype was isolated craniofacial FD. Thirty-three patients presented during adulthood (51%).

**Table 1:** Clinical and demographic characteristics of patients with confirmed FD/MAS

FD/MAS characteristics	58
Type of FD/MAS, no of patients (%)	
MFD	15 (25.8)
CFD	19 (32.7)
PFD	10 (17.2)
MAS	13 (22.4)
FD/MAS with Myxomas	1 (1.7)
Median age at presentation in years (range)	20 (0–72)
MFD	28 (6–65)
CFD	39 (8–72)
PFD	12 (3–61)
MAS	6 (1–20)
Endocrinopathies, *N* (%)	11 (19)
GIPP	8 (13.7)
GH excess	3 (5)
Hyperprolactinemia	3 (5)
Hyperthyroidism	2 (3.4)
FGF23-induced Hypophosphataemia	1 (1.7)

*FD/MAS* fibrous dysplasia/McCune-Albright syndrome, *MFD* monostotic fibrous dysplasia, *CFD* craniofacial fibrous dysplasia, *PFD* polyostotic fibrous dysplasia, MAS McCune-Albright syndrome, *GIPP* gonadotropin-independent precocious puberty, GH growth hormone

#### Diagnosis Confirmation

Responders were asked about the methods used to confirm the diagnosis; multiple combinations were possible. Among the 15 patients with MFD, diagnosis was confirmed using radiological findings in 13 patients (86%), history and clinical findings in 12 (80%), blood biochemistry in nine (60%) and biopsy in five (33%). A GNAS mutation was detected in two patients (13%).

For the 10 patients with PFD, radiological findings were used in all 10 patients (100%), followed by history and clinical findings in nine (90%), blood biochemistry in five (50%) and biopsy in two (20%).

Among the 13 patients with MAS, diagnosis was confirmed based on history and clinical findings in all patients (100%), blood biochemistry in 12 (92%), radiological findings in 11 (85%), a positive GNAS mutation test in five (38%) and biopsy in three (23%).

For the 19 patients with isolated CFD, radiological findings were used in all cases (100%), history and clinical findings in 18 (94%), blood biochemistry in 15 (79%), biopsy in four (21%) and a positive GNAS mutation test in one patient (5%).

#### Disciplines Involved in the Care Pathway

Endocrinology was the first contact discipline at the participating centres in 26/58 cases (45%), followed by orthopaedic surgery in 15/58 cases (26%) and otorhinolaryngology in six (10%). Disciplines involved in the care pathway included endocrinology in 34/58 cases (59%), ophthalmology in 20 cases (35%) and orthopaedic surgery in 17 (29%) (Table 2). The number of specialists involved ranged from one to eight, with a median of two. Most patients with MFD were seen by two specialists (73%) while patients with CFD were seen by two (47%), five (26%) and four (21%). Patients with PFD were seen by three specialists in 30% of the cases and by one, two and six in 20%, respectively. Patients with MAS were seen by four specialists (31%), two (23%), one (15%), four (15%), six (0.7%) and seven (0.7%).

**Table 2:** First contact disciplines at the reference centre and other disciplines involved in the care of FD/MAS. Endocrinology and paediatric endocrinology are presented together

	MFD (*n* = 15)		CFD (*n* = 19)		MAS (*n* = 13)		PFD (*n* = 10)		MZ (*n* = 1)	
Specialist	MFD First	MFD Other	CFD First	CFD Other	MAS First	MAS Other	PFD First	PFD Other	MZ First	MZ Other
Endocrinology	4	10	9	10	9	7	3	7	1	
Orthopaedic surgery	8	1	1	1	2	13	4	4		1
Ear, nose and throat		1	5			6	1	1		
Craniofacial surgery	2						1			
Neurosurgery			1		1	1		2		
Maxillofacial surgery			1	1				2		
Ophthalmology		3	1	5		7		5		
General Paediatrics		1			1	1				
Genetics		1					1			
Dentist		1		3		4		3		1
Dermatologist		2				1				
Rheumatology		1						1		
Radiologist		1								
Neurologist		2		1		1		1		
Psychologist						2				
Gynaecologist		1				1				
General surgeon				1				1		1
Cardiology		1								1

*MFD* monostotic fibrous dysplasia, *PFD* polyostotic fibrous dysplasia, *MAS* McCune-Albright syndrome, *CFD* craniofacial fibrous dysplasia, *MZ* fibrous dysplasia with myxoma

#### General Measurements and Characteristics

##### Skeletal Involvement

Of 58 patients, 44 (76%) had skeletal involvement without any extraskeletal features. Of these 44, 19 had CFD (43%), 15 had MFD (34%) and 10 had PFD (23%).

Fourteen of 58 (24%) patients had extraskeletel components. Of these, five had only extraskeletal features, specifically endocrinopathies, as the sole clinical manifestation of the disease. Within this group, 13 (92%) had MAS and one had FD with myxomas.

##### Endocrine Testing

Endocrine evaluation was performed in 62% (36/58) of patients across seven centres. All patients underwent assessment by an endocrinologist either as first contact specialist in 19 cases (53%) or specialist involved in the care in 17 cases (47%). Of these 36 patients, 14 (39%) had PFD, 17 (47%) had MFD and 5 (14%) did not have any skeletal involvement. Of the 58, 11 (19%) had abnormal endocrine findings and therefore were classified as MAS.

##### McCune-Albright Syndrome

Of 13 cases of MAS, one had MFD (8%), seven had PFD (54%) including three with craniofacial involvement, and five (38%) without skeletal lesions. Endocrinopathies were present in 11 patients. The most prevalent abnormality was gonadotropin-independent precocious puberty (GIPP) in eight patients (62%), followed by growth hormone (GH) excess and hyperprolactinaemia in three patients, respectively (23%), and thyrotoxicosis in two (15%) (Table 1).

Of eight patients with GIPP, six had GIPP as their only endocrine abnormality, one in combination with GH excess, thyrotoxicosis and hyperprolactinaemia, and one in combination with GH excess and hyperprolactinaemia. One patient had GH excess in combination with hyperprolactinaemia and one patient had thyrotoxicosis only.

Serum phosphate was normal in 54 (93%) patients. Fibroblast growth factor 23 (FGF23) was measured in 20 (34%) patients. Bone markers were measured in 50 patients (86%) in five centres and these included type 1 procollagen amino terminal peptides (P1NP) (*n* = 31), alkaline phosphatase (*n* = 30), c-terminal telopeptide of type I collagen (CTX-I) (*n* = 25), and osteocalcin (*n* = 4) (Table 3).

**Table 3:** Frequency of use of different diagnostic techniques across centres

Centre	No. of cases	HCF	Blood biochemistry	GNAS mutation present	Radiology	Biopsy	Serum phosphate	FGF23	P1NP	ALP	CTX-I	Osteocalcin
A	43	40 (93)	35 (81)	5 (11)	41 (95)	9 (30)	39 (91)	18 (41)	28 (65)	26 (60)	22 (51)	2 (5)
B	5	3 (60)	0	1 (20)	4 (80)	1 (20)	5 (100)	0	0	0	0	0
C	5	5 (100)	3 (60)	1 (20)	4 (80)	2 (40)	5 (100)	0	0	0	0	0
D	2	2 (100)	1 (50)	0	2 (100)	1 (50)	2 (100)	0	0	1 (50)	0	1
E	2	2 (100)	2 (100)	0	2 (100)	1 (50)	2 (100)	0	0	0	0	0
F	1	1 (100)	1 (100)	1 (100)	1 (100)	0	1 (100)	0	1 (100)	1 (100)	1 (100)	1 (100)

*HCF* history and clinical findings, *P1NP* type 1 procollagen amino terminal peptides, *ALP* alkaline phosphatase, *CTX-I* c-terminal telopeptide of type I collagen, *FGF23* fibroblast growth factor 23

Standardised questionnaires were used routinely in 21/58 patients (36%) being the Brief Pain Inventory (BPI) the most commonly used in 21 cases (34%) followed by EQ-5D in four (7%), and Patient-Reported Outcomes Measurement Information System (PROMIS) in two (3%).

Nineteen patients had received intravenous bisphosphonates (32.7%), 25 received calcium/vitamin D3 supplements (43.1%), five received oral bisphosphonates (8.6%), two received denosumab (3.4%), two received calcitriol/alfacalcidol (3.4%), and one phosphate (1.7%).

Of these 58 patients, 52 had been entered in the EuRREB Core Registry by the time of the survey. Participation in other registries was not reported.

#### Module Development

The results of this survey provided us with detailed information on how patients with FD/MAS are approached, diagnosed, treated, and followed up at the reference centres. In combination with the expertise of the study group, these results were used to develop a set of 88 variables, 86 clinician-reported and 2 patient-reported variables, covering several aspects of the care of patients with FD/MAS (Online resource 2).

Clinician-reported variables were categorised in nine domains: anthropometry, a basic disease domain, disease characteristics, MAS, craniofacial FD, FD with myxoma, intraductal papillary and mucinous neoplasms of the pancreas (IPMN) and therapy. (Table 4). Additionally, the study group issued a recommendation for the use of two patient-reported outcomes measures: EQ-5D, Brief Pain Inventory-Short Form.

**Table 4:** Different domains in the module and number of variables included in each domain

Domain	No. of variables
Anthropometry	6
Basic disease	10
Disease characteristics Musculoskeletal Endocrinopathies Bone and mineral metabolism Neoplasms	16
McCune-Albright syndrome	6
Craniofacial FD	15
FD with Myxoma	11
IMPN	10
Therapy	10

*FD* fibrous dysplasia; *IPMN* intrapapillary mucinous neoplasm

After its launch in April 2022, the module was updated by the expert group based on clinical needs and further refinement. In the orthopaedic domain, variables collecting detailed information on fibrous dysplasia related fractures were added. The variables on visual outcomes in the CFD domain were also expanded, including now specific data on nerve palsy, new visual complaints and underlying eye disease (Online Resource 2). This was done with experts in the field and discussed within the FD/MAS condition-specific module study group.

## Discussion

In this survey among expert centres throughout Europe, we enquired about patients diagnosed with FD/MAS. The results show great variability in the diagnostic and care pathways of these patients. This inconsistency is not surprising as this is a rare condition and until 2019 no guidelines or care pathway had been published [16]. In addition, the disease presents with a broad clinical spectrum, and patients are managed by many different medical specialties.

Most patients required three methods to confirm the diagnosis with variable combinations. In the case of MFD and CFD, only 33 and 25% of patients, respectively, underwent a biopsy. However, especially in CFD, a biopsy is recommended to reach the diagnosis if there is any doubt, as the radiological features overlap with other conditions that require a different approach [17]. In PFD and MAS, the clinical, biochemical and radiological picture will almost always be sufficient to make a diagnosis [16]. However, we did observe that in 20% of the PFD cases and 23% of the MAS cases, invasive procedures like biopsies were performed.

In our study, 38% of patients with MAS did not have FD as a component of the disease. This contrasts with reports in the literature, which acknowledge that MAS can occur without skeletal lesions but generally describe FD as its main component. [1].

The broad clinical spectrum and complexity of the condition is also shown by the variability in the number of specialists involved, where most MFD patients are seen by two specialists while most MAS patients are seen by five.

Overall, the endocrinologist (adult/paediatric) was the most frequent first contact specialist at the reference centre (53%). Endocrinology was also the most frequent discipline involved in the care (60%) across all FD subtypes. This contrasts with what has been reported by Song et al., where the endocrinologist was not a frequent specialist during the diagnostic journey [15].

The current best practice guidelines recommend the assessment of pain using a visual analogue scale (VAS) 0–10 and the BPI in adults or Wong Baker Facies in children [16]. Although the BPI was the most reported questionnaire, it was only used in one-third of the patients, all from the same centre and including two children. The use of VAS and Wong Baker Facies was not reported but the percentage of patients who were below the age of 18 was only 41.3%. Furthermore, assessing pain using these tools was not routinely performed in patients treated with bisphosphonates or denosumab since only 57% reported the use of these questionnaires. This is remarkable since denosumab is advised as last resort in patients with bisphosphonate-resistant pain or progressive lesion growth and bisphosphonates are recommended for pain treatment not to prevent lesion growth [9, 16].

The results of this survey show that the care of patients with FD/MAS is indeed performed by healthcare professionals with expertise in different fields of medicine. Therefore, a multidisciplinary registry/condition-specific module is necessary. Also, these results highlight the importance of collecting data in a homogeneous way, most important when studying rare conditions where the number of patients is scarce, and the data of each patient are highly valuable.

There are some limitations of our study to consider when interpreting the results. Although the response rate for this survey was high, three-quarters of the cases were reported by two centres in the Netherlands posing the risk of geographical and centre bias. Furthermore, these centres provide tertiary care which might not represent the practice in other centres caring for patients with FD/MAS. However, the results of the survey and proposed data elements were discussed within the FD/MAS study group and adjusted after feedback; therefore, we consider that this did not influence the module development.

Following the collection of the survey responses, the registries study group on FD/MAS integrated a multidisciplinary team of healthcare professionals and representatives of patient advocacy groups and brought together their expertise to develop a condition-specific dataset. The project benefited from the differences in roles and knowledge areas, for example, the initiative to collecting information on IPMNs or craniofacial FD, where the approach posed in some cases a clinical dilemma. The variables were integrated in the EuRREB Core Registry (Core Registry—EuRREB (EuRRECa and EuRR-Bone) as a condition-specific module which was launched in April 2022. After obtaining informed consent from the patient, the healthcare professional may enter the data which include a list of core data fields and FD/MAS-specific data to characterise the natural history of this group of conditions. Additionally, PROMs to assess pain and quality of life can be completed within the platform by healthcare professionals and patients with access [18]. The Core Registry platform currently holds 670 cases of FD/MAS from nine centres in eight countries. Our aim is to perform a first interim analysis by the beginning of 2025 and expert centres throughout the world are invited to participate. International disease registries can collect data to fill knowledge gaps about this rare group of conditions, providing insights into the diagnostic pathway, current care and patient monitoring. Furthermore, patient registries can support benchmarking in clinical practice, helping to identify the areas for improvement and implement best practices.

## Supplementary Information

Below is the link to the electronic supplementary material.

Supplementary file1 (PDF 165 KB)

Supplementary file2 (PDF 118 KB)
