# Supplementary material for: Developing a Standardised Dataset for Natural History Studies in Fibrous Dysplasia/McCune-Albright Syndrome
Source: Calcif Tissue Int. 2025 May 2;116(1):68. doi: 10.1007/s00223-025-01379-5 (PMC12048454; doi:10.1007/s00223-025-01379-5)
Supplement: Supplementary file 1 — Supplementary file1 (PDF 165 KB) [file 223_2025_1379_MOESM1_ESM.pdf]

e-Reporting for Rare Conditions

Bone: Fibrous Dysplasia/McCune Albright Syndrome survey

1. Further information about this survey

Aim of this survey

To collect routine clinical data for quality assurance and for understanding the diagnostic processes and clinical outcome of cases with FD/MAS conditions in children and in adults that have been reported on EuRR-Bone's e-reporting platform (e-REC).

Governance

The EuRRECa project launched in 2018 and the EuRR-Bone project launched in 2020, includes e-REC which is approved by the UK research ethics service to collect non-personally identifiable clinical data and does not require individual patient consent. To participate in e-REC, the clinician has to register; a list of participating centres and further information is available at https://eurreca.net/e-rec/. Participating centres are advised to obtain local approvals at their own centre. The survey questionnaire utilises Webropol, a secure on-line tool that is endorsed and supported by NHS Greater Glasgow & Clyde and NHS Scotland. All information provided will be kept in compliance with the General Data Protection Regulation (GDPR 2016/679) and the UK Data Protection Act (2018). The EuRRECa and EuRR-Bone project teams will have access to the complete dataset and will provide data to research teams following approval by the Data Access Committee. These data will only be shared with investigators following the approval of the clinician who is responsible for the patient.

Further contact

It is possible that the EuRRECa/EuRR-Bone project team may contact you again to check the data submitted and to provide you with further reports of the data.

Natasha Appelman Dijkstra - EuRR-Bone project lead
Faisal Ahmed – EuRRECa project lead
Ana Priego - EuRRECa/EuRR-Bone clinical fellow

FD/MAS group
Roland Chapurlat - Lyon
Kassim Javaid - Oxford
Maartje E. Meier - Leiden
Diana Ovejero Crespo, Hospital del Mar
Lothar Seefried - Wuerzburg
Daniele Tessaris - Turin

info@eurreca.net
EuRR_bone@lumc.nl

I confirm that I have read the above information and I am happy to proceed to the survey *

- ◯ Yes
- ◯ No

2. e-REC ID *

____

3. Clinician responsible for the patient (for any outputs from this work, this clinician will be contacted)

____

4. E-mail address of clinician responsible for the patient

____

5. Diagnosis status

- ◯ Suspected
- ◯ Confirmed
- ◯ Excluded

6. Age at presentation in years

____

7. Type of FD/MAS: Select all that apply

- ☐ MFD
- ☐ PFD
- ☐ MAS
- ☐ Mazabraud
- ☐ CFD

8. How was diagnosis confirmed (tick all that apply)

- ☐ History and clinical findings
- ☐ Blood biochemistry
- ☐ Radiologic findings
- ☐ GNAS mutation present
- ☐ Biopsy

9. Which specialist was the first point of contact in the reference center?

- ○ Dermatologist
- ○ Endocrinology
- ○ ENT
- ○ Nurse specialist
- ○ Orthopedic surgery
- ○ Rheumatology
- ○ Ophthalmologist
- ○ Other: ____________________

10. Which other disciplines are involved in the care of the patient? (select all that apply)

- ☐ Dentist
- ☐ Dermatologist
- ☐ Endocrinology
- ☐ ENT
- ☐ Nurse specialist
- ☐ Orthopedic surgery
- ☐ Rheumatology
- ☐ Ophthalmologist
- ☐ Other: ____________

11. When did the dentist get involved?

- ○ At diagnosis (within the first week)
- ○ Within the first 3 months

12. When did the dermatologist get involved?

- ◯ At diagnosis (within the first week)
- ◯ Within the first 3 months

13. When did the endocrinologist get involved?

- ◯ At diagnosis (within the first week)
- ◯ Within the first 3 months

14. When did the ENT get involved?

- ◯ At diagnosis (within the first week)
- ◯ Within the first 3 months

15. When did the nurse specialist get involved?

- ◯ At diagnosis (within the first week)
- ◯ Within the first 3 months

16. When did the orthopedic surgeon get involved?

- ◯ At diagnosis (within the first week)
- ◯ Within the first 3 months

17. When did the rheumatologist get involved?

- ◯ At diagnosis (within the first week)
- ◯ Within the first 3 months

18. When did the opthalmologist get involved?

- ◯ At diagnosis (within the first week)
- ◯ Within the first 3 months

19. When did the other specialist get involved?

- ◯ At diagnosis (within the first week)
- ◯ Within the first 3 months

20. Was serum phosphate low in this patient?

- ◯ Yes
- ◯ No
- ◯ Unknown

21. What is the renal tubular reabsorption of phosphate?

- ◯ Normal
- ◯ Low (adjusted for age). Value: ____________________

22. Were endocrinopathies tested?

- ◯ Yes
- ◯ No
- ◯ Not known

23. Endocrinopathies results

- ☐ Results not known
- ☐ No abnormalities
- ☐ Abnormalities
- ☐ Gonadotropin-independent Precocious puberty
- ☐ GH excess
- ☐ Thyrotoxicosis
- ☐ Hyperprolactinemia
- ☐ Neonatal hypercortisolism
- ☐ Other: ____________

24. Were bone markers increased?

- [ ] Yes
- [ ] No
- [ ] No not measured

25. Which bone markers were measured?

- [ ] Alkaline phosphatase
- [ ] P1NP
- [ ] CTx
- [ ] Osteocalcin
- [ ] FGF23
- [ ] Other: ____________

26. Was the patient on bone regulating medication? select all that apply

- [ ] Bisphosphonate oral
- [ ] Bisphosphonate IV
- [ ] Denosumab
- [ ] Calcitriol/alfacalcidol
- [ ] Phosphate
- [ ] Calcium/d3 supplements
- [ ] Calcitonin

27. Do you use standardised questionnaires regularly? Select all that apply

- ○ Yes
- ○ No

28. Which standardised questionnaires do you use? (Select all that apply)

- [ ] EQ5D
- [ ] SF-36
- [ ] BPI
- [ ] Pain detect
- [ ] PROMIS
- [ ] WHODAS

☐ IPQ

☐ other ______________

29. Has the patient been added into the following registries

☐ EuRRECa/EuRR-Bone Core Registry

☐ Other, please specify: ______________

Manuscript title: Developing a Standardised Dataset for Natural History Studies in Fibrous Dysplasia/McCune-Albright Syndrome

Journal: Calcified Tissue International and Musculoskeletal Research Journal

Author list: Ana Luisa Priego Zurita, Oana O Bulaicon, Jillian Bryce, Nerea Arrieta, Magdalena Caballero Campos, Mariya Cherenko, Gaby Doxiadis, Corinna Grasemann, M Kassim Javaid, Helen McDevitt, Stijn W van der Meeren, Diana Ovejero Crespo, Luisa de Sanctis, Lothar Seefried, Annemarie A Verrijn Stuart, Daniele Tessaris, Pieter Bas de Witte, Roland Chapurlat, S Faisal Ahmed, Natasha M Appelman-Dijkstra

Corresponding author:
Natasha M. Appelman-Dijkstra
Department of Internal Medicine, Division of Endocrinology
Leiden University Medical Center
Albinusdreef 2, Leiden, Postbox 9600, 2300 RC, The Netherlands
N.M.Appelman-Dijkstra@lumc.nl
