# Supplementary material for: Developing a Standardised Dataset for Natural History Studies in Fibrous Dysplasia/McCune-Albright Syndrome
Source: Calcif Tissue Int. 2025 May 2;116(1):68. doi: 10.1007/s00223-025-01379-5 (PMC12048454; doi:10.1007/s00223-025-01379-5)
Supplement: Supplementary file 2 — Supplementary file2 (PDF 118 KB) [file 223_2025_1379_MOESM2_ESM.pdf]

Online resource 2

Domain	Variable name	Updated version
**Anthropometry**		
	Height (cm)	
	Height, SDS	
	Weight (kg)	
	Weight, SDS	
	BMI kg/m^2	
	BMI, SDS	
**Basic disease module**		
	How was diagnosis reached?	
	Disease components	
	Treatment history	
	Other surgeries	
	Current therapy	
	Disease related hospital admissions since last visit	
	Pregnancy	
	Number of deliveries	
	Is patient alive?	
	Cause of death if applicable	
**Disease characteristics**		
Musculoskeletal		
	Fractures due to fibrous dysplasia	
	Limb deformity due to fibrous dysplasia	
	Scoliosis due to fibrous dysplasia	
	Pain at fibrous dysplasia site	
Endocrinopathies		
	McCune/Albright related endocrinopathy	
Bone and mineral metabolism		
	Alkaline phosphatase	
	CTX-I	
	Hypophosphatemia, serum confirmation	
	PINP	
	Hypophosphatemia, TmP/GFR confirmation	
	TmP/GFR value	
	Intact FGF-23 value (pg/ml)	
	C-terminal FGF-23 value (U/ml)	
	PTH	
Neoplasms		
	Neoplasms	
	Other neoplasm	

**McCune-Albright syndrome**		
	Type of endocrinopathy	
	Age at presentation of neonatal hypercortisolism if applicable	
	Age at presentation of precocious puberty if applicable	
	Age at presentation of hyperthyroidism if applicable	
	Age at presentation of growth hormone excess if applicable	
	Age at presentation of hyperprolactinemia if applicable	
**Craniofacial FD**		
	Side of craniofacial lesion	
Visual		
	Best corrected Visual Acuity	
	Was OCT (optical coherence tomography) performed?	
	Visual field left status	
	Visual field right status	
	Nerve palsy left	
	If yes, specify nerve	✓
	Nerve palsy right	
	If yes, specify nerve	✓
	New visual complaints	
	Underlying eye disease	✓
Ear, nose and throat		
	ENT complaints	
	Hearing loss type right	
	Hearing loss severity right	
	Hearing loss type left	
	Hearing loss severity left	
	Paranasal sinus involvement left	
	Paranasal sinus involvement right	
**Therapy**		
Past Therapy		
	FD related medication	
	Indication for FD related medication	
	Endocrinopathy related medication	
	Endocrinopathy related, other therapies	
Current therapy		
	FD related medication	
	Indication for FD related medication	
	Endocrinopathy related medication	
Adverse events (current or past therapy related)		
	Has the patient developed FD related medication adverse events?	
	Please indicate the medication	
	Adverse event	

**IPMN**		
Imaging		
	Imaging technique	
	Cyst diameter	
	Is the cyst located in the head of the pancreas?	
	Is there an enhancing solid component within the cyst?	
	Presence of thickened/enhancing cyst walls?	
	Presence of non-enhancing mural nodule?	
Clinical findings		
	Symptoms related to IPMNs	
Laboratory values		
	CA 19-9 U/ml	
Complications		
	IPMN related complications	
Treatment		
	IPMN treatment	
**Fibrous dysplasia with myxoma**		
Diagnosis		
	How was the myxoma diagnosed?	
	If it differs from what specified in the Core Data please explain	
Myxoma related clinical findings		
	Number of myxomas	
	Location of myxoma(s)	
	Symptoms	
Myxoma related complications		
	Myxoma related complications	
Myxoma related treatment		
	Myxoma related treatment	
	Were there any surgery related complications?	
	Surgery related complications	
Myxoma cellularity		
	Cellularity based on pathology	
**Orthopaedic**		
Fractures	Fibrous dysplasia related fractures	✓
	Number of fractures	✓
	Year of fracture	✓
	Fracture mechanism	✓
	Fracture location	✓
Surgery		
**Patient-reported outcomes**		
	How many health professionals do you/the patient see?	

	Which health professionals do you/the patient see regularly?	
**Patient-reported outcome measures (PROMs)**		
	EQ-5D	
	BPI-SF	
	NPSI	✓

Online resource 3. Set of clinician and patient-reported variables. Clinician-reported variables were categorised in different domains. A basic disease module collecting general information on the condition was followed by domains covering specific areas of the care, disease subtypes, and disease course. ALP: alkaline phosphatase; BMI: body mass index; BPI-SF: brief pain inventory short form; CTX-I: c-terminal telopeptide of type I collagen; FD: fibrous dysplasia; FGF23: fibroblast growth factor 23; GH: growth hormone; IPMN: intrapapillary mucinous neoplasm intrapapillary mucinous neoplasm; NPSI: neuropathic pain symptom inventory; P1NP: type 1 procollagen amino terminal peptides; PTH: parathyroid hormone; SDS: standard deviation score; TmP/GFR: ratio of tubular maximum reabsorption of phosphate to glomerular filtration rate

Title: Developing a Standardised Dataset for Natural History Studies in Fibrous Dysplasia/McCune-Albright Syndrome

Journal: Calcified Tissue International and Musculoskeletal Research Journal

Author list: Ana Luisa Priego Zurita, Oana O Bulaicon, Jillian Bryce, Nerea Arrieta, Magdalena Caballero Campos, Mariya Cherenko, Gaby Doxiadis, Corinna Grasemann, M Kassim Javaid, Helen McDevitt, Stijn W van der Meeren, Diana Ovejero Crespo, Luisa de Sanctis, Lothar Seefried, Annemarie A Verrijn Stuart, Daniele Tessaris, Pieter Bas de Witte, Roland Chapurlat, S Faisal Ahmed, Natasha M Appelman-Dijkstra

Corresponding author:
Natasha M. Appelman-Dijkstra
Department of Internal Medicine, Division of Endocrinology
Leiden University Medical Center
Albinusdreef 2, Leiden, Postbox 9600, 2300 RC, The Netherlands
N.M.Appelman-Dijkstra@lumc.nl
